# Identification of neutralizing nanobodies protecting against poxvirus infection

**DOI:** 10.1038/s41421-025-00771-7

**Published:** 2025-03-25

**Authors:** Xuehua Yang, Li Guo, Huarui Duan, Miao Fan, Fengwen Xu, Xiaojing Chi, Shengnan Pan, Xiuying Liu, Xinhui Zhang, Peixiang Gao, Fangyuan Zhang, Xinyi Wang, Fei Guo, Jiwan Ge, Lili Ren, Wei Yang

**Affiliations:** 1 Key Laboratory of Pathogen Infection Prevention and Control (Ministry of Education), National Institute of Pathogen Biology, Chinese Academy of Medical Sciences & Peking Union Medical College, Beijing, China; 2 NHC Key Laboratory of Systems Biology of Pathogens, National Institute of Pathogen Biology, Chinese Academy of Medical Sciences & Peking Union Medical College, Beijing, China; 3 NHC Key Laboratory of System Biology of Pathogens and Christophe Mérieux Laboratory, National Institute of Pathogen Biology, Chinese Academy of Medical Sciences & Peking Union Medical College, Beijing, China; 4 State Key Laboratory of Respiratory Health and Multimorbidity, Chinese Academy of Medical Sciences & Peking Union Medical College, Beijing, China

**Keywords:** Immunology, Biological techniques

## Abstract

An outbreak of mpox has triggered concerns regarding the adequacy of intervention strategies. Passive immunity conferred by neutralizing antibodies exhibits potential in the prophylaxis and treatment of orthopoxvirus infections. Despite this, the investigations of effective antibody therapeutics have been hindered by the varied nature of orthopoxvirus envelope proteins and the intricate mechanisms underpinning viral invasion. Our study involves the production of six mpox virus (MPXV) envelope proteins, which are relatively conservative and considered to play a role in the neutralization process. We employed a synthetic nanobody (Nb) library to derive a broad array of specific Nbs against these viral proteins. We identified a cross-reactive Nb, termed M1R-01, which targets the M1R protein and effectively neutralizes both vaccinia virus (VACV) and MPXV. Notably, the M1R-01-based antibody strategy provided optimal protection against a lethal VACV challenge in mice. Additionally, we determined the crystal structure of the M1R–Nb complex, uncovering novel binding attributes of M1R-01 and detailed conformational epitope information. This work provides a promising candidate for the therapy and prophylaxis of orthopoxvirus infections.

## Introduction

Within the schema of the *Poxviridae* family, the *Orthopoxvirus* genus contains several pathogens of medical significance. These include the causative agents for smallpox (variola virus, VARV), mpox (formerly monkeypox, MPXV), cowpox (CPXV), and vaccinia virus (VACV)^1^. Notably, VACV has played a pivotal role in the eradication of smallpox, following its deployment in vaccination campaigns. MPXV, initially isolated from monkeys in 1958, is the etiological agent of human mpox, a zoonotic disease first identified in central Africa in 1970^2^. A new round outbreak of mpox occurred in May 2022, precipitating a marked increase in global cases. As of April 30th, 2024, a cumulative total of 97,208 laboratory-confirmed cases of mpox, including 186 deaths, were reported by the World Health Organization (WHO). In August 2024, the WHO re-designated the mpox outbreak as a “public health emergency of international concern”. The clinical manifestations of mpox share similarities with smallpox, which has prompted the hypothesis that existing smallpox vaccines may confer protection against MPXV infections or reduce their severity. However, the termination of the smallpox vaccination program in 1980 left an entire generation of individuals unexposed to both MPXV and VARV, making them vulnerable to infection.

Recent clinical studies have shown that MPXV infection in humans elicits robust serum IgG responses against multiple viral membrane proteins^3^. Orthopoxviruses possess a diverse array of membrane proteins that are incorporated into the viral membrane and function in multiple ways. There are two distinct forms of infectious particles: extracellular enveloped virions (EEVs) and intracellular mature virions (IMVs)^4^. An EEV particle consists of an IMV-like particle surrounded by an additional membrane containing at least six viral proteins unique to EEVs. Poxviruses have at least four membrane proteins (D8, H3, A26, and A27) responsible for cell binding and ten proteins for membrane fusion, forming a complicated entry-fusion complex (EFC)^4^. These membrane proteins display significant sequence and functional conservation across orthopoxviruses, making them important targets for vaccine development and antiviral therapy.

Among MPXV membrane proteins, A29L shares homology with VACV A27 and is located on the surface of IMV. It is responsible for binding heparin to the cell surface, regulating membrane fusion, and mediating the transport of IMV to form EEV^5^. A35R is homologous to VACV A33R and serves as an envelope glycoprotein of EEV, playing a pivotal role in effective EEV and being required for the formation of actin-containing microvilli and cell–cell spread of virion^6^. M1R, a myristate-modified surface membrane protein conserved across IMV, is involved in viral particle assembly and the stimulation of immune protection. B6R is a palmitoylated glycoprotein present in EEV. It is indispensable for several critical steps of the viral life cycle, including the envelopment of the virus, the formation of filopodia, and the dissolution of the EEV membrane immediately prior to virus entry into the host cell^7^. H3L is located on the IMV and interacts with heparan sulfate moieties on the cell surface, facilitating virus–host interaction and potentially influencing the efficiency of viral entry. E8L serves as a glycosaminoglycan (GAG)-binding protein within the IMV. It binds to chondroitin sulfate on the cell surface and is essential for the adsorption of the IMV to the host cell, a crucial step in the viral entry process. These viral proteins have been identified as important targets for the generation of neutralizing antibodies or as constituent elements of vaccines^5,8–14^.

The 2022 mpox outbreak has been noted to primarily affect individuals who are concurrently living with human immunodeficiency virus (HIV)^15^. Within this population, the efficacy of active immune defense mechanisms is notably attenuated, which potentially hinders their capacity to mount an effective response against the MPXV infection. Antibody-based therapy is considered as a potential treatment option for severe mpox. Vaccinia immune globulin (VIG) could be utilized in mpox therapy though systematical clinical cohort investigation is not available^16^. In animal models, passive administration of several monoclonal antibodies has been shown to be protective against mpox^5,17–19^. Currently, some monoclonal antibodies with high neutralizing efficacy^5,20^, such as 7D11, are generated in mice through VACV infection or immunization with recombinant membrane proteins^19^. Human-sourced antibodies targeting A33, A27, B5, and L1 have been isolated and demonstrated promising in vitro or in vivo neutralizing efficacy^21,22^. However, the majority of reported antibodies are complement-dependent and effective protection was achieved through a “cocktail” strategy that combines multiple antibodies targeting different membrane proteins.

Nanobodies (Nbs), also named VHHs or single-domain antibodies, represent the smallest naturally occurring antibody fragments, originating from the heavy-chain-only immunoglobulins of the *Camelidae* family. Nbs are distinguished by their compact structure, ease of production via prokaryotic expression systems, high resilience in adverse environmental conditions, superior aqueous solubility, and the ability to modulate their half-life^23^. In light of the current limitations associated with poxvirus-neutralizing antibodies and considering the distinctive attributes of Nbs, the present investigation employed a previously established fully synthetic Nb library platform to screen Nbs that target multiple MPXV membrane proteins. An Nb with affinity for the M1R protein was identified and found to exhibit superior neutralization efficacy against both VACV and MPXV. A comprehensive examination of its binding epitope, the underlying mechanism of action, and its capacity to elicit protective effects in vivo were also conducted.

## Results

### Conservation analysis and preparation of the selected MPXV membrane proteins

**Fig. 1:**
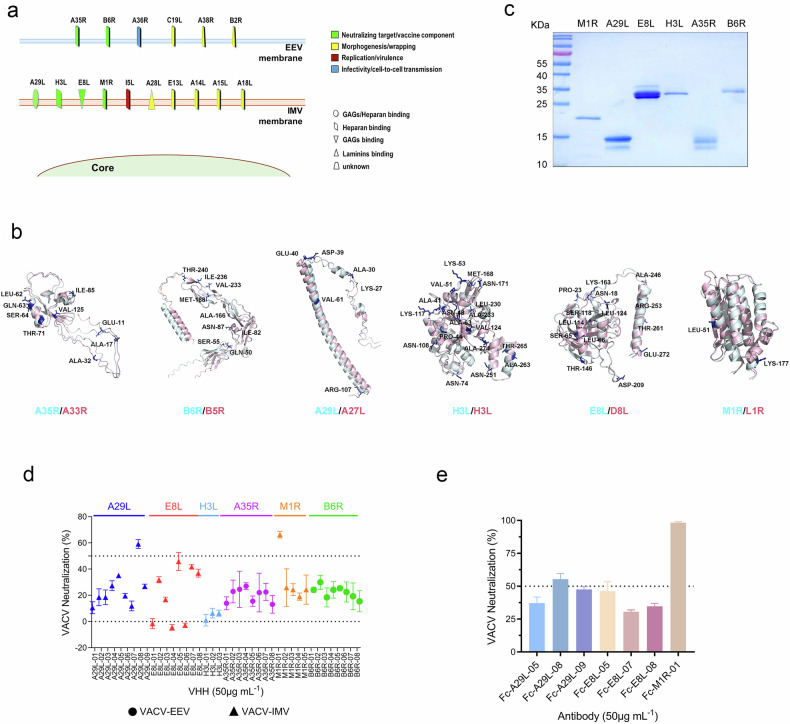
Screening and evaluation of Nbs directed against six MPXV membrane proteins. **a** The MPXV particle contains approximately 20 membrane proteins that are distributed across the intracellular mature virus (IMV) or extracellular enveloped virus (EEV), with their respective functions indicated. **b** Six of these membrane proteins, which serve as targets for neutralization or are components in vaccine formulations, were selected as antigens for Nb screening. A comprehensive structural comparison was conducted on the extracellular domains of these proteins from MPXV and VACV. The different amino acid sites in VACV are labeled. **c** SDS-PAGE and Coomassie Brilliant Blue staining of the purified viral membrane proteins. **d** Forty-one Nbs were obtained and their neutralizing capabilities were assessed against the IMV or the EEV of VACV-WR. Each symbol represents the mean ± standard deviation (SD) of the triplicate of neutralizing efficacy. **e** The selected Nbs were fused with mouse IgG-Fc and their neutralizing potency was reassessed at a concentration of 50 μg/mL against IMV of VACV-WR. The bars represent the mean ± SD of the neutralization results from triplicate experiments.

Poxviruses are acknowledged as among the most intricate viruses described, distinguished by their unique bilayer membrane structure, accompanied by ~20 membrane-associated proteins that encircle either EEV or IMV (Fig. 1a). Grounded in extant research regarding the functions of viral membrane proteins, six of them were identified as prime targets for neutralizing antibodies or as key immunodominant components for vaccine development. These MPXV candidates include the IMV membrane proteins A29L, H3L, E8L, and M1R, along with the EEV membrane proteins B6R and A35R.

To analyze the conservation of these proteins, a structural comparison was performed on the extracellular domains of the aforementioned six membrane proteins from MPXV and VACV (Fig. 1b). Overall, the comparative analysis has elucidated a high degree of structural conservation among these predicted proteins, albeit with variability in the degree of sequence similarity among them. Within the context of the antigens selected, M1R exhibits two discrepant amino acids among its total of 181, whereas the A29L protein presents with six differing amino acids out of 110, nine out of 129 for A35, 15 out of 274 for E8L, nine out of 279 for B6, and a total of 20 out of 282 for H3L. Collectively, these six proteins evidenced a significant degree of conservation across both virus species, underscoring their potential importance in vaccine development and antiviral strategies.

To conduct a systematic exploration of protective antigens and the selection of neutralizing antibodies against MPXV, we constructed expression plasmids that encoded the ectodomains of the above six membrane proteins. This effort was guided by the nucleotide sequence of a recent MPXV isolate, MPXV_USA_2022_MA001 (GenBank accession number: ON563414.3). The authenticity and integrity of the purified recombinant proteins were verified through SDS-PAGE (Fig. 1c). Additionally, commercial antibodies were utilized for antigen identification through enzyme-linked immunosorbent assay (ELISA) (Supplementary Fig. S1). The purification process successfully yielded all six proteins with levels of purity that were suitable for subsequent Nb screening.

### Screening of neutralizing Nbs targeting MPXV membrane proteins

Leveraging our previously established fully synthetic and diversified Nb phage display library platform^24,25^, we conducted targeted screenings of Nbs against the six membrane proteins of MPXV. Following four rounds of reciprocal biopanning coupled with phage ELISA validation, we isolated a collection of Nb binders. This collection comprised nine Nbs specific for A29L, five for M1R, three for H3L, and eight each for E8L, A35R, and B6R. These Nbs were produced in *Escherichia coli* and purified with one-step nickel affinity chromatography (Supplementary Fig. S2). The sequences of screened Nbs are listed in Supplementary Table S1.

We evaluated the neutralization efficacy of these Nbs using VACV (strain WR) EEV or IMV. The majority of the Nbs exhibited neutralization efficacies of less than 30% against VACV. However, the neutralizing efficacy of eight Nbs was ~40% or higher, including A29L-05, A29L-08, and A29L-09 targeting the A29L protein, along with E8L-02, E8L-05, E8L-07, and E8L-08 targeting the E8L protein, and M1R-01 targeting the M1R protein (Fig. 1d). Notably, M1R-01 exhibited the highest neutralization activity, nearly reaching 70%, followed by A29L-08, which demonstrated activity of ~60%. Furthermore, Surface Plasmon Resonance (SPR) was used to assess the binding affinity between each Nb and its corresponding target protein. The affinity data are listed in Supplementary Table S2. The majority of these Nbs exhibited a relatively strong affinity for their targets. Specifically, more than half of these Nbs demonstrated an equilibrium dissociation constant (*K*_D_) within the order of several to tens of nanomoles per liter (nM).

In general, the neutralizing capabilities of the obtained Nbs were suboptimal, with the exception of M1R-01 and A29L-08. The prevailing view within the field is that most neutralizing antibodies against poxviruses function complement-dependently or may lose activity in the absence of complement^5,14,21,22,26^. Moreover, considering the small size of Nbs and their limited spatial occupancy, it was deemed necessary to modify these entities. We hope to augment their neutralizing potential by engineering them into a larger format through the fusion of mouse IgG2a-Fc. In total, seven Fc-fused Nbs were obtained. The affinity of these Fc-fused Nbs was markedly enhanced, with an increase ranging from several to several dozenfold, primarily characterized by an improvement in the dissociation rate constant *K*_off_ (data not shown). However, the results presented in Fig. 1e indicated that six out of the seven Fc-fused Nbs demonstrated neutralizing activities similar to their monovalent predecessors, with no discernible increase in activity attributable to Fc fusion. Exceptionally, Fc-M1R-01 exhibited a significant boost, achieving over 98% neutralization at a concentration of 50 μg/mL. Due to its outstanding neutralizing efficacy, subsequent efforts were concentrated on the characterization of M1R-01.

### The multivalent Nbs demonstrate improved affinity and neutralization

**Fig. 2:**
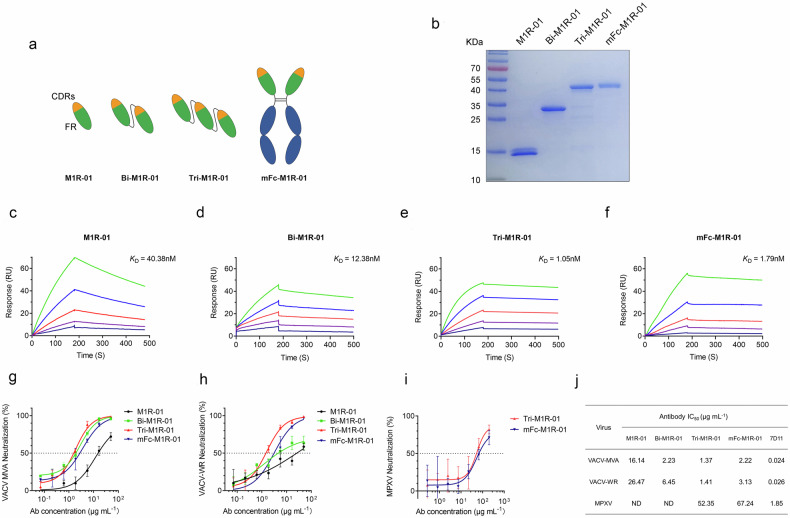
Generation and validation of multivalent and Fc-fused M1R-01. **a** Structural representation of the monovalent (M1R-01), bivalent (Bi-M1R-01), trivalent (Tri-M1R-01), and Fc-fused (mFc-M1R-01) forms of M1R-01. **b** Purified Nbs were verified by SDS-PAGE and Coomassie Brilliant Blue staining. **c**–**f** Affinities of M1R-01 (**c**), Bi-M1R-01 (**d**), Tri-M1R-01 (**e**), and Fc-M1R-01 (**f**) were assessed using SPR. **g**, **h** Neutralizing activities of these four variants of M1R-01 against IMV of modified vaccinia Ankara (MVA) (**g**) or VACV-WR (**h**) were evaluated. **i** The neutralizing activity of Tri-M1R-01 and Fc-M1R-01 against MPXV. Data were expressed as the mean ± SD and are derived from one of three independent experiments. **j** Summary of the IC_50_ values obtained from the neutralization assays, with data representing the mean of three separate experiments.

To augment the potency and expand the utility of M1R-01, we engineered bivalent and trivalent variants of the Nb, named Bi-M1R-01 and Tri-M1R-01, respectively. The structural configurations of these constructs are illustrated in Fig. 2a, where the Nbs are interconnected via a flexible (G_4_S)_3_ linker. The purified antibodies were analyzed by SDS-PAGE (Fig. 2b), which confirmed the expected molecular weights of ~30 kDa for the bivalent and 45 kDa for both the trivalent and mFc-fused forms. SPR analysis was employed to assess the affinity of these antibody forms for interaction with the recombinant M1R protein (Fig. 2c–f). The data revealed a gradual enhancement in affinity, with the *K*_D_ values for Tri-M1R-01 and mFc-M1R-01 determined to be 1.05 and 1.79 nM, respectively. This represents an ~20–40-fold increase in affinity compared to that of the monovalent M1R-01.

Neutralization activities revealed a consistent and notable enhancement with the transition from monovalent to multivalent (Fig. 2g, h). The IC_50_ values were computed and presented in Fig. 2j, which represented the mean IC_50_ values determined from the triplicate measurements for each Nb construct. The monovalent M1R-01 exhibited a mean neutralizing IC_50_ of roughly 20 μg/mL against both strains. In contrast, the Bi-M1R-01 and Tri-M1R-01 displayed significantly higher neutralizing activities. Fusion with the mouse IgG-Fc resulted in an IC_50_ values ~2–3 μg/mL, which represented a tenfold improvement in neutralizing efficacy and was slightly inferior to the Tri-M1R-01. Notably, both Tri-M1R-01 and the mFc-M1R-01 variants achieved neutralization levels exceeding 90% against VACV. To account for the potential influence of varying IgG-Fc formats on the Nb’s neutralizing capacity, the fusion Nb constructs were engineered to contain different Fc fragments, derived from human IgG1, mouse IgG1, and IgG2a. The outcomes of the plaque reduction assays targeting the two vaccinia virus strains indicated that the Nbs, regardless of the Fc types, retained comparably effective neutralizing capabilities (Supplementary Fig. S3).

MPXV was used to further evaluate the neutralizing capacity of mFc-M1R-01 and Tri-M1R-01. The results indicate that both antibodies achieved a 50% inhibition of MPXV at concentrations around 60 μg/mL, with a maximum neutralization efficacy of 80% at the highest concentration tested (Fig. 2i). In comparison to VACV, the neutralizing capacity against MPXV reduced 20 to 40-fold. To determine whether this reduced sensitivity to neutralizing antibodies is a general characteristic of MPXV or due to other specific reasons, we conducted additional experiments using 7D11, a previously characterized mouse monoclonal antibody with a strong neutralizing effect on VACV. 7D11 was found to achieve over 90% neutralization of VACV at a concentration of ~1 μg/mL, with an IC_50_ value as low as 0.024 μg/mL (Supplementary Fig. S4). However, when tested against MPXV, 7D11 demonstrated a significant decrease in efficacy, resulting in only an ~80% reduction in virus titer at a concentration of 10 μg/mL. Moreover, it took more than 1.8 μg/mL of 7D11 to inhibit MPXV plaque formation by 50% (Supplementary Fig. S4), which represents an ~70-fold decrease in efficacy when compared to VACV. These findings suggest that, compared to VACV, MPXV is inherently less susceptible to antibody neutralization. This finding has also been observed in other studies^21^.

### Nasal administration of Tri-M1R-01 protects VACV infection in mice

**Fig. 3:**
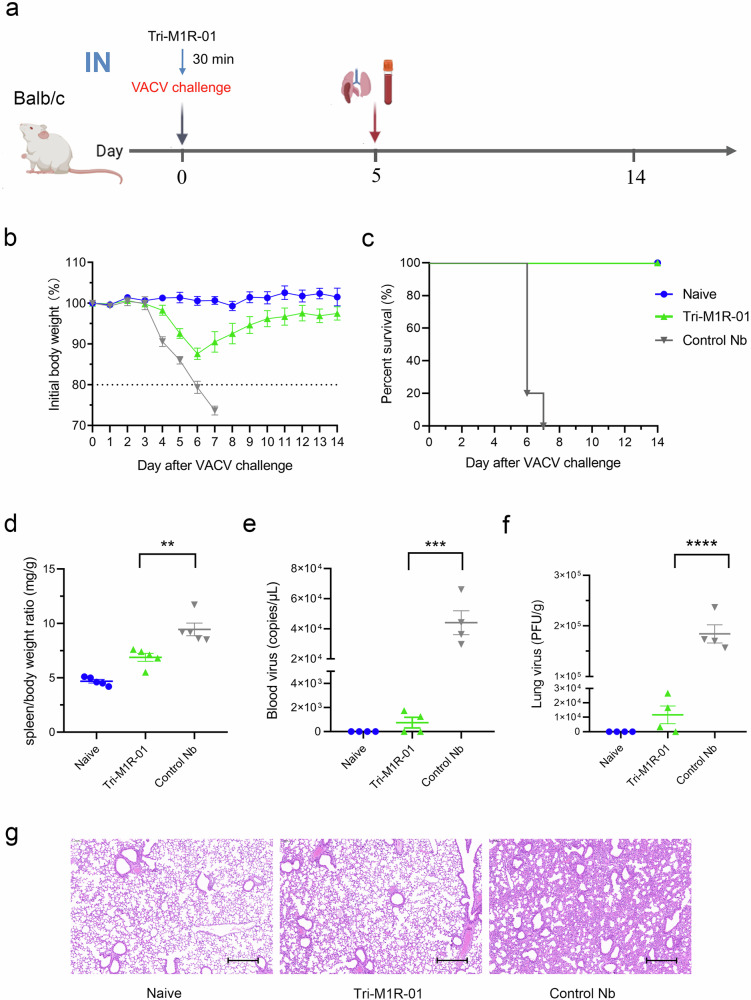
Prophylactic effects of Tri-M1R-01 against VACV infection via intranasal administration in mice. **a** Experimental design: Balb/c mice were administered intranasally (IN) with 10 mg/kg of Tri-M1R-01 or an anti-SARS-CoV-2 Nb (NB10) as a control. Thirty minutes after the administration, mice were IN challenged with a lethal dose of VACV and survival was monitored. Five days post-infection, lungs, spleens, and blood were collected for subsequent analysis. **b** Time course of body weight changes in Balb/c mice (*n* = 5 per group). **c** Survival curves of mice (*n* = 5 per group). **d** Spleen-to-body weight ratio (mg/g), *n* = 5, bars indicate the mean ± SEM. **e**, **f** Virus titers in the blood (**e**) and lungs (**f**) were determined. Bars represent the mean ± SEM of mice (*n* = 4). **g** The Hematoxylin and Eosin (H&E) staining of lung sections from each group. Scale bars, 300 µm. The data presented in this figure are derived from one of two independent experiments, with 8–10 mice per group. A one-way ANOVA test was employed for significance analysis. Statistical significance was shown as *P < 0.05, **P < 0.01, ***P < 0.001, ****P < 0.0001.

Neutralizing antibodies can elicit passive immune intervention against viral infections through both prophylactic and therapeutic mechanisms. Given the high stability and relatively low production cost of Nbs, we designed a prophylactic protection experiment in mice (Fig. 3a). The Tri-M1R-01 was instilled into the nasal cavities of mice prior to challenge with a lethal dose of VACV-WR. Mice treated with Tri-M1R-01 exhibited significant protection against mortality. The control group experienced over a 20% loss of its original body weight by days 5–6, reaching the criteria for death determination. In contrast, mice receiving Tri-M1R-01 showed some weight loss during days 4–6 and then rapidly recovered and eventually returned to their original weight, with no mortality observed (Fig. 3b, c).

Upon necropsy on day 5 post-infection, marked splenomegaly, and significant weight loss were observed in the mice infected with VACV-WR, resulting in an elevated spleen-to-body weight ratio. In contrast, the spleen-to-body weight ratio of the mice treated with Tri-M1R-01 was markedly lower than that of the control Nbs, indicative of reduced spleen weights and comparatively stable body weight (Fig. 3d). Furthermore, the levels of viral load in blood and lung tissues of the mice treated with Tri-M1R-01 were significantly lower than those in the control Nb-treated group (Fig. 3e, f), suggesting an effective inhibition of viral replication and dissemination. Furthermore, in the histopathological analysis of mouse lung tissues, we observed a significant reduction in lung tissue lesions following nasal administration with Tri-M1R-01 (Fig. 3g).

### Intraperitoneal injection of Fc-M1R-01 protects mice from fatal VACV challenge

**Fig. 4:**
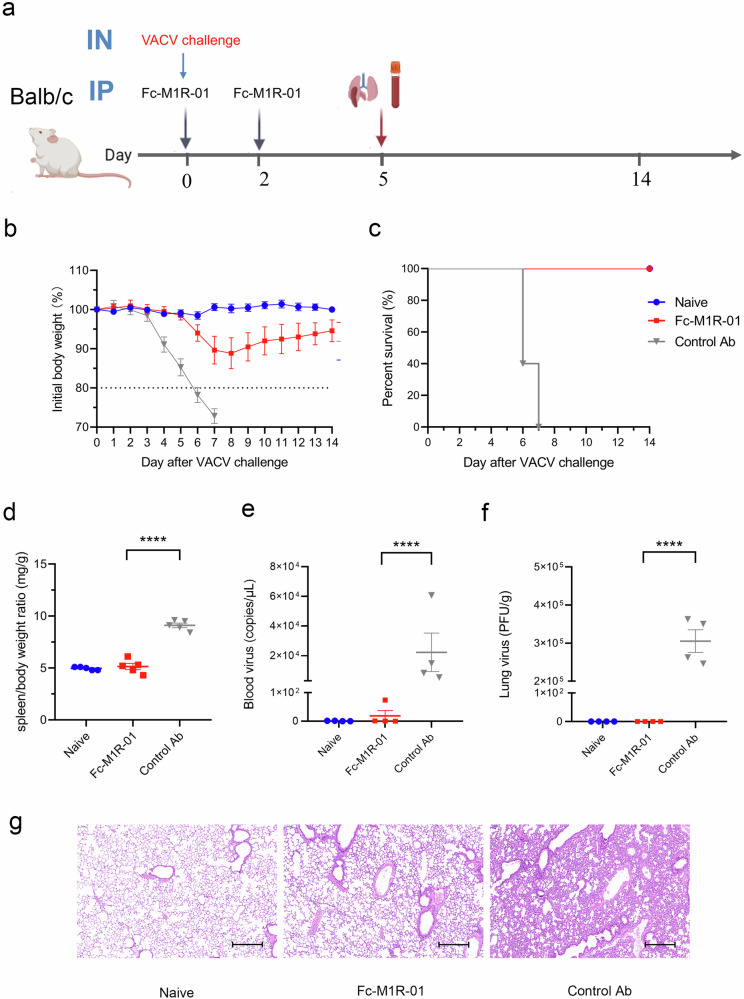
Protective effects of Fc-M1R-01 against VACV challenge via intraperitoneal injection in mice. **a** Experimental design: Balb/c mice were intranasally (IN) challenged with a lethal dose of VACV, following which they were administered intraperitoneally (IP) with 10 mg/kg of Fc-M1R-01 or an anti-GP73 antibody served as a control at days 0 and 2. The mice were then monitored for survival. At 5 days post-infection, lungs, spleens, and blood were collected for subsequent analysis. **b** Time course of body weight changes in Balb/c mice (*n* = 5 per group). **c** Survival curves of mice (*n* = 5 per group). **d** Spleen-to-body weight ratio (mg/g), *n* = 5; bars indicate the mean ± SEM. **e**, **f** Virus titers in the blood (**e**) and lungs (**f**) were determined. Bars represent the mean ± SEM of mice (*n* = 4). **g** The H&E staining of lung sections from each group. Scale bars, 300 µm. A one-way ANOVA test was employed for significance analysis. Statistical significance was shown as *P < 0.05, **P < 0.01, ***P < 0.001, ****P < 0.0001.

In addition to evaluating the prophylactic efficacy, we also investigated the therapeutic potential of M1R-01 through intraperitoneal injection (Fig. 4a). For this purpose, the human IgG1-Fc fusion antibody of M1R-01 was generated for the in vivo study. Considering that its half-life might be short, the Fc-M1R-01 was administered to mice on days 0 and 2. After the VACV challenge, mice treated with control antibodies exhibited a precipitous decline in body weight beginning on day 4, ultimately leading to the lethal endpoint. In contrast, mice treated with Fc-M1R-01 maintained a stable body weight throughout the study period and exhibited overall good health conditions (Fig. 4b, c). Analysis of the spleen-to-body weight ratio revealed that there was no significant difference between the Fc-M1R-01-treated mice and the non-infected control group, suggesting that Fc-M1R-01 provided excellent protection against the VACV infection (Fig. 4d).

This protective effect was further corroborated by blood virus titration results: approximately 10^4^ copies/μL of the virus were detected in the control Ab group, whereas almost no virus was detected in the Fc-M1R-01-treated mice (Fig. 4e). Similarly, lung tissue virus detection demonstrated a minimal presence of VACV in the Fc-M1R-01 treatment group, indicating a profound reduction in viral load in the respiratory tract (Fig. 4f). Similar to the nasal administration group, histopathological analysis of mouse lung tissue also revealed a significant reduction in lung tissue lesions following intraperitoneal administration of Fc-M1R-01 (Fig. 4g). These findings collectively demonstrate the therapeutic efficacy of Fc-M1R-01 in inhibiting VACV replication and disease progression, thereby conferring a significant level of protection to the treated mice.

### Crystal structure of M1R-01 in complex with M1R

**Fig. 5:**
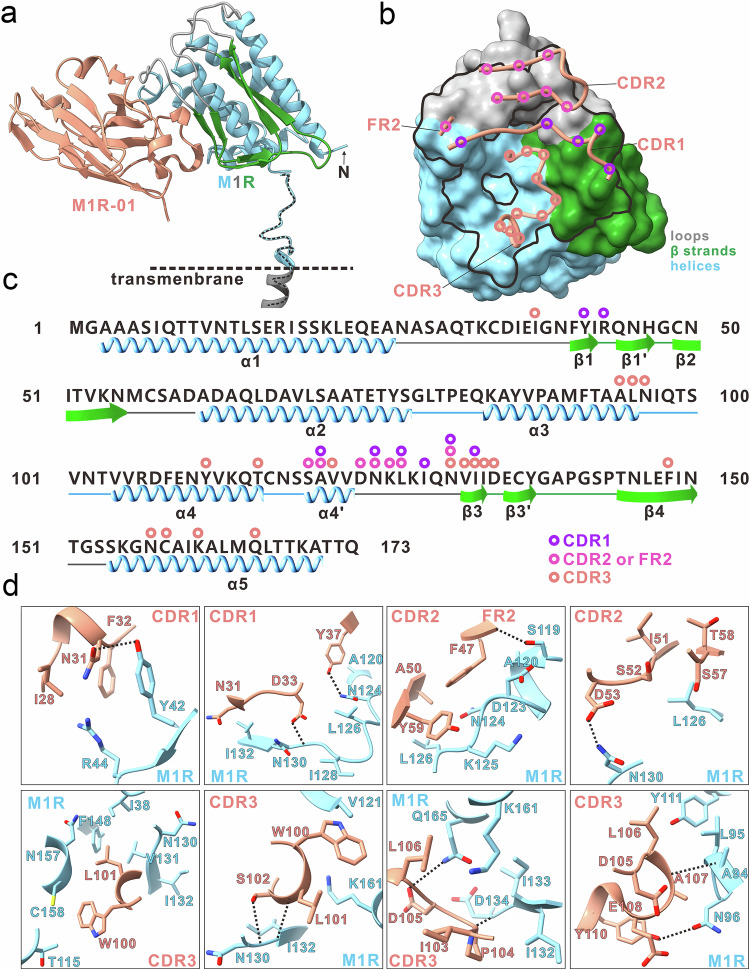
Crystallographic structure of the Nb M1R-01 complexed with M1R. **a** Cartoon representation of the crystal structure showing the Nb M1R-01 in complex with its target protein, M1R. The ectodomain of M1R is depicted based on the predicted full-length structure by AlphaFold2. M1R is depicted in three distinct colors: sky blue for α-helices, dark gray for loops, and forest green for β-strands. The Nb is shown in dark salmon. The transmembrane domain of M1R is demarcated by black dashed lines. **b** Close-up view of the surface of M1R interacting with M1R-01. The epitope of M1R-01 on M1R is footprinted with a black line. The corresponding CDRs are shown with the interacting residues highlighted with colored circles. **c** Sequence and secondary structure of M1R. The locations of helices and β-strands based on the M1R structure are shown, with the circles indicating the contacting residues on M1R. **d** The binding interface between the M1R and M1R-01. The hydrogen bonds are shown as black dashed lines.

To delineate the specific epitope recognition mechanism, we determined the complex structure of M1R and M1R-01 at a resolution of 2.59 Å. The quality of the data analysis is indicated by the final R_work_ and R_free_ statistics of 23.91 and 25.95%, respectively (Fig. 5a and Supplementary Table S3). The orientation of M1R, supposed with its stem region perpendicular to the viral surface, allows M1R-01 to make a diagonal approach at ~45° angle relative to the viral membrane surface (Fig. 5a). M1R-01 employs its three complementarity determining regions (CDRs), with particular emphasis on the elongated CDR3, to project into the groove that is formed by the clustered α helices (α4, α5, and α3), β strands (β3 and β4), and the loop region connecting α4 and the β3 on M1R (Fig. 5b, c).

The specific binding orientation of M1R-01 yielded an unexpectedly high buried surface area (BSA) of 1129.5 Å², in contrast to the BSA of neutralizing antibodies 7D11, M12B9, A138, and A129 (data unpublished), which were 825, 943, 798, and 622 Å², respectively. This indicates that the interface between M1R and M1R-01 undergoes extensive interactions (Supplementary Fig. S5), particularly within the CDR3 region (W100–A110) (Fig. 5c, d and Supplementary Table S4). The CDR3 region alone accounts for more than half of the total interactions. The corresponding CDR3-interacting residues on M1R are clustered within a focused epitope, encompassing α3 (A94–L95), α4 (Y111 and T115), α5 (N157, C158, K161, and Q165), α4’ (V121) the loop connecting α4’ and β3 (N130), β3 (V131–I133), β4 (F148), along with the sporadic residues I38, N96, D134 (Fig. 5c, d and Supplementary Table S4). In addition, the CDR1 (I28, N31–D33, and Y37), CDR2 (A50, I51, S52, D53, S57, T58, and Y59), and FR2 (F47) regions primarily establish a limited number of contacts with α4’, and the loop between α4’ and β3 (Fig. 5c, d and Supplementary Table S4). Among these interactions, a total of 11 hydrogen bonds were identified, with six involving the CDR3, three in CDR1, one in FR2, and one in CDR2 (Fig. 5d and Supplementary Table S4). These findings underscore the distinctive binding characteristics of M1R-01 with its target antigen.

### The binding epitope of M1R-01 is distinct from 7D11 and conserved in orthopoxvirus

**Fig. 6:**
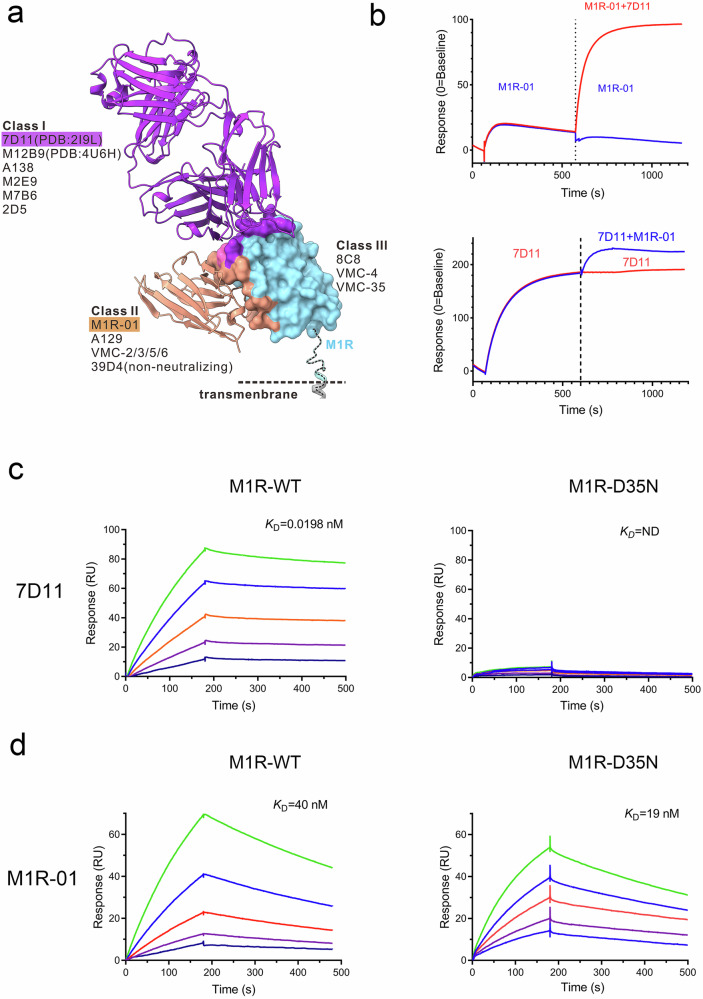
Characterization of the binding epitopes for M1R-01 and 7D11. **a** Classification of antibodies targeting M1R/L1R. **b** SPR-based epitope competition assay to assess the binding interference between M1R-01 and 7D11. One antibody was used to pre-block the immobilized antigen and allowed to reach saturation prior to the flow of the other antibody across the sensor surface. Individual binding events are color-coded. **c**, **d** The affinity of wild-type M1R (left) and a D35N mutant M1R (right) for mouse monoclonal antibody 7D11 (**c**) and Nb M1R-01 (**d**) was determined using SPR.

To date, several antibodies targeting the L1R/M1R have been isolated and investigated. However, the lack of comprehensive structural investigations has prevented the formation of a consensus group among these antibodies. In this study, we categorize these antibodies into three distinct classes based on their distinct binding epitopes (Fig. 6a). The class of 7D11, including A138, M12B9, M2E9, M7B6^18^, and 2D5^27^, are all neutralizing antibodies and bind around Asp35, which we have named class I. The binding epitope for class I antibodies is situated on the opposite side of the viral surface. Class II antibodies, represented by A129, approach M1R from the side and encompass both neutralizing (M1R-01, A129, VMC-2/3/5/6)^26^ and non-neutralizing antibodies (39D4)^18^. We assign M1R-01 to class II based on the partial overlap of its epitope with that of class II antibodies, which are known to bind to a linear epitope within the range of 120–130 amino acids on the L1R antigen. It is important to note that although M1R-01 belongs to class II, it recognizes a conformational rather than a linear epitope, which exhibits enhanced specificity. Furthermore, a series of other non-neutralizing antibodies, including 8C8^18^, VMC-4, and VMC-35^26^, do not compete with antibodies from Class I and II and are accordingly grouped into Class III. This classification system provides a structured framework for understanding the diverse binding strategies of antibodies against the M1R antigen.

The above epitope information was corroborated by epitope competition assays conducted between M1R-01 and 7D11, which revealed no overlap in their binding epitopes (Fig. 6b). The SPR data demonstrated that the binding of one antibody to M1R did not preclude the subsequent binding of the other, regardless of the order of their attachment. Furthermore, we engineered a mutant form of the M1R protein, M1R-D35N, to investigate the role of Asp35 in the interaction between 7D11 and M1R. Consistent with prior studies^18,28^ this mutation completely abolished the robust binding between 7D11 and M1R, whereas M1R-01 maintained its high affinity for both the wild-type M1R and the D35N mutant (Fig. 6c, d).

**Fig. 7:**
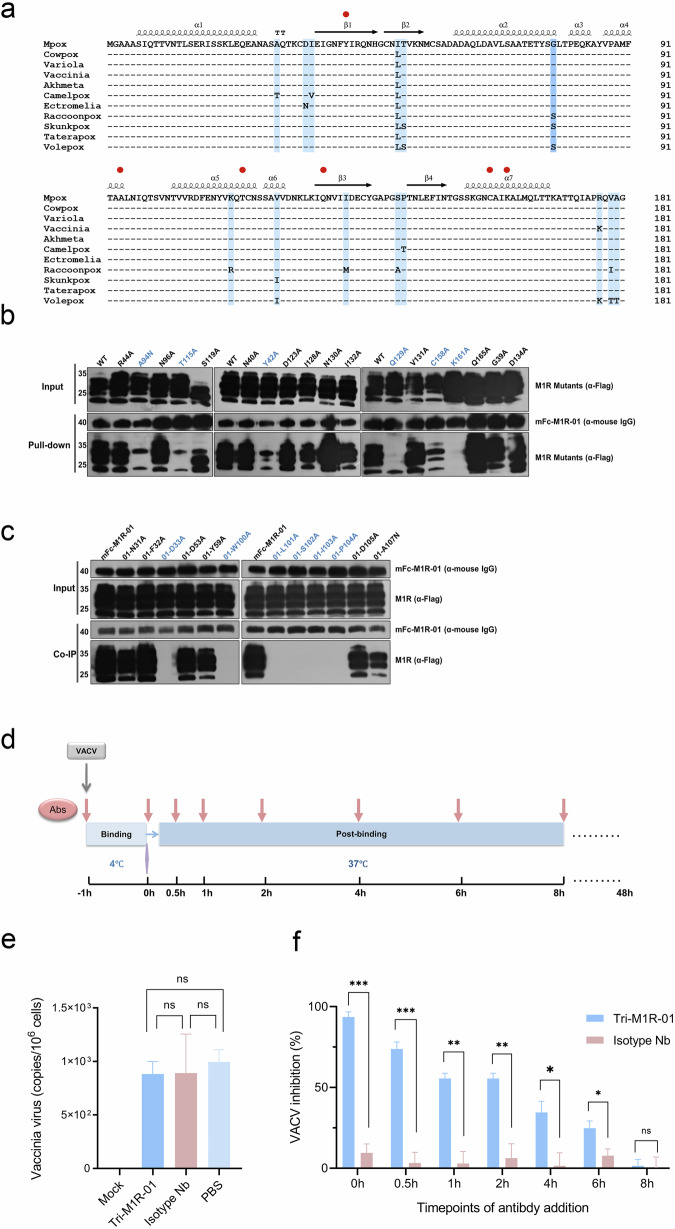
M1R-01 recognizes the conserved epitopes across orthopoxvirus and plays a neutralizing role in the post-binding stage. **a** Alignment of M1R extracellular domain in 11 viral strains within *Orthopoxvirus*. Different colors indicate the degree of conservation at different sites: clear is completely consistent, followed by light blue, and the site with the greatest difference is represented by the deeper blue. Above the sequence, the secondary structures, such as α helices and β sheets that correspond to the amino acid positions, are represented. The red dots indicate the key residues on the M1R protein that are associated with the binding to M1R-01 (corresponding to the results in **b**). **b** Pull-down assay to determine the critical residues in M1R for association with Fc-M1R-01. The key sites are labeled in blue. **c** Co-IP assay to determine the key amino acids in M1R-01 for association with the antigen M1R. **d** Schematic diagram of experimental determination of the virus entry steps affected by M1R-01. For the binding step, the VACV was mixed with Tri-M1R-01, added into the pre-cooled cells, and allowed for incubation at 4 °C for 1 h. Then, the cells were washed with cold PBS and harvested for qRT-PCR analysis of viral DNA copies. For the post-binding step, the VACV was first incubated with the target cells at 4 °C for 1 h, and then moved to 37 °C. Tri-M1R-01 or control isotype Nb was added at different timepoints, and plaques were counted at 48 h post-infection. **e** Quantification of the cell-bound virus in the presence or absence of antibodies. Mock means cells without the addition of VACV and antibodies. Isotype Nb is a non-relevant trivalent Nb as the negative control. Bars indicate mean ± SEM of triplications. **f** Inhibition of VACV plaque formation by antibodies added at different post-binding timepoints. Bars indicate mean ± SEM of triplications. *t*-test was used for comparisons between groups. Statistical significance was shown as *P < 0.05, **P < 0.01, ***P < 0.001.

To explore the conservation of the epitope recognized by M1R-01 across different poxvirus species, we performed an amino acid sequence alignment of the M1R homologs from 11 orthopoxvirus strains (Fig. 7a). This analysis revealed that, while there is a high degree of sequence similarity among these strains, there are variations at certain amino acid positions, most notably at the 78th position, where either Glycine or Serine can be found. Additionally, mutations were identified at 13 amino acid sites, including positions 30, 35, 36, 51, 52, 113, 121, 133, 143, 144, 177, 179, and 180. Among the four human pathogenic strains, MPXV, VACV, VARV, and CPXV, only two amino acids differ at positions 78 and 177. Notably, our findings indicate that M1R-01 does not interact with any of these mutated sites. Based on these observations, we suggest that M1R-01 exhibits broad-spectrum antiviral potential against a wide range of orthopoxvirus members.

### M1R-01 plays a neutralizing role in the post-binding stage

At the biochemical level, to further elucidate the molecular mechanisms underlying the key interactions between M1R and M1R-01, we developed a series of mutants for both proteins. Amino acid residues were systematically replaced with Alanine, and the original Alanine residues were substituted with Asparagine. We employed co-immunoprecipitation (co-IP) and pull-down techniques to identify several crucial residues involved in the interaction. As shown in Fig. 7b, mutations at multiple sites on M1R resulted in a decrease or complete abrogation of its interaction with M1R-01. Notable mutations include A94N, T115A, Y42A, Q129A, C158A, and K161A. Conversely, mutations in M1R-01, such as D33A, W100A, L101A, S102A, I103A, and P104A, also led to a loss of antigen–antibody associations (Fig. 7c). Importantly, with the exception of D33A, the other critical residues are situated within the CDR3 region of M1R-01. This finding is in agreement with the crystal structural data, which suggests that the CDR3 region is the primary interacting loop responsible for the specific binding between M1R and M1R-01.

Finally, we sought to elucidate which steps during virus entry are blocked by M1R-01. Simply, the VACV entry was divided into two distinct steps, the initial virus binding phase and the subsequent post-binding phase (Fig. 7d). Our data suggest that the addition of M1R-01 during the virus attachment stage did not result in a substantial attenuation of virus abundance on the cell surface (Fig. 7e). Conversely, the application of M1R-01 during the post-binding timepoints engendered a detectable reduction in viral plaque formation, most pronounced when the antibody intervention was administered within the first 30 min (Fig. 7f). This corroborated the existence of a heightened neutralizing efficacy within this timeframe. The delayed M1R-01 addition, particularly beyond the 2-h mark, manifested a marked diminution in neutralizing potency.

## Discussion

The research presented here culminated in the production of six conserved membrane proteins from the MPXV, which were screened through a systematic Nb library and led to the discovery of an M1R-specific Nb, designated M1R-01, capable of cross-reacting with both VACV and MPXV. The protective efficacy of M1R-01 was explicitly demonstrated in a murine model, where it conferred robust survival benefits against lethal VACV infections. Comprehensive structural analyses were conducted to elucidate the binding characteristics and epitope information of M1R-01. These findings highlighted the unique attributes of M1R-01, underscoring its potential as a prophylactic and therapeutic candidate for combating infections caused by orthopoxviruses. The structural insights obtained are instrumental in laying the groundwork for the future development of novel immunological strategies against these diseases.

The intricacies of the orthopoxvirus membrane structure are pivotal to the virus’s capacity to invade host cells, yet they present significant challenges for the development of antiviral drugs that target the viral entry process. This complexity is manifested in a double membrane architecture emanating from the trans-Golgi network, a complicated membrane protein composition, and glycosylation. The identification of essential membrane protein targets for the stimulation of highly effective neutralizing antibodies is, therefore, a pivotal step in the fight against these infections. Our synthetic Nb library platform is suited to this task due to its exceptional diversity, operational efficiency, and high-throughput capabilities. Our screenings have revealed that Nbs targeting MPXV IMV (anti-H3L, anti-A29L, anti-E8L, and anti-M1R) and EEV (anti-B6R and anti-A35R) proteins exhibit varying degrees of neutralizing potential. However, the neutralizing efficacy of Nbs targeting distinct membrane proteins exhibited notable variation. This observation aligns with earlier reports that highlighted the dependence of complement for optimal neutralization activity^12^ and that antibodies specific to the M1R may employ a distinct molecular mechanism compared to other neutralizing antibodies. It also suggests that the M1R protein may be targeted by non-classical antibody biomolecules, such as Nbs or peptides, offering new avenues for the development of therapeutics against orthopoxviruses.

Passive immunotherapy, exemplified by vaccinia immune globulin intravenous (VIGIV), serves as a crucial line of defense against mpox^29^. This therapeutic approach can be particularly beneficial for individuals with weakened immune systems, such as those with advanced HIV infection, who may lack the capacity to mount an effective antibody response against the virus. To date, no therapeutic monoclonal antibodies have been sanctioned for the treatment of poxvirus infections. Currently, investigations in both vaccine (mRNA or subunit vaccines) design and antibody-based immunoprotection predominantly entail the integration of multiple membrane protein targets^14,21,30,31^, which may primarily arise from the notion that neutralizing antibodies directed at a single epitope may not suffice to ensure substantial protection. The study presented here demonstrates that M1R-01, a novel Nb, can potently neutralize both VACV and MPXV. Remarkably, in murine lethal infection models, M1R-01 provided effective protection against VACV infection. Nevertheless, the therapeutic potential of M1R-01 following MPXV exposure remains speculative. This uncertainty is due, in part, to the notable difference in the susceptibility of MPXV to neutralizing antibodies when compared to VACV. It has indeed been documented in several studies that the MPXV exhibits reduced sensitivity to neutralizing antibodies compared to the VACV^21,22^. Notably, M1R, which shares a high degree of homology with L1R, stands out as the most conserved protein across the entire orthopoxvirus family (Figs. 1b, 7a). Our analysis of different clades of MPXV has revealed that M1R remains conserved and has not undergone mutation. We hypothesize that the observed phenomenon may be attributed to several factors: (1) variations in the quantity and proportion of membrane proteins present on the two viruses, (2) differences in the infection and replication efficiencies between MPXV and VACV, and (3) potentially more efficient utilization of direct cell-to-cell transmission by MPXV as opposed to VACV. Actually, a similar reduction in neutralizing antibody sensitivity has been noted in the cowpox virus. Additionally, the requirement for a higher antibody dosage to potentially enhance therapeutic efficacy further complicates the assessment of M1R-01’s utility post-MPXV infection.

Currently, the majority of mouse strains are refractory to MPXV infection. Although CAST/Eij mice can experience transient infection, the MPXV (clade-2 strain) is sufficiently mild to preclude lethality and does not produce overt symptoms such as weight loss. The only observed effects are a minor swelling of the spleen and a relatively low serum viral load. Consequently, there is no stable and reliable rodent model for MPXV available at this time. Recently, several publications have documented the use of Balb/c mice for MPXV infection^20,32^. We remain committed to exploring potential mouse models for MPXV and will continue these efforts in our future work. Future research must focus on elucidating the precise mechanism(s) of action and the efficacy of M1R-01 in the context of MPXV neutralization and therapeutic intervention.

In this study, we also elucidated the epitope recognition profile of M1R-01 through the detailed analysis of the crystal structure of the antigen–antibody complex. Concurrently, a comprehensive review and categorization of existing antibodies that target the M1R/L1Rs were conducted. The epitopes recognized by these antibodies were categorized into three distinct classes. Notably, antibodies binding to the distal epitope of M1R (Class I), which is distant from the viral membrane, such as mAb 7D11, exhibit potent neutralizing capabilities. The epitope targeted by M1R-01 has been allocated to Class II, and it does not share overlap with the 7D11 epitope yet maintains a significant degree of conservation. This systematic categorization will purportedly expedite the future development of neutralizing antibodies directed against M1R, encompassing the design of bispecific or multispecific neutralizing antibodies. The L1R/M1R protein is essential for virus entry and membrane fusion; however, the precise mechanism underlying this process remains elusive, including the identification of their host–virus interaction partners and the critical functional domains involved. Following the report of several L1-neutralizing antibodies, we have postulated that the head region of L1/M1R, also known as the Class I epitope in this work, plays a pivotal role in triggering the key biological events required for virus entry, including membrane fusion. Adjacent to the Class I epitope is the Class II epitope, which, due to its close proximity, results in antibodies targeting the Class II region being less effective than those targeting the Class I region, mainly due to weak steric hindrance. Given the smaller size of Nbs, we recognize the need for genetic engineering to increase their size, which may enhance their neutralizing activity.

This research holds the potential to inform the advancement of therapeutic strategies targeting M1R, which is pivotal in the context of mpox pandemics. Collectively, the present study has successfully identified the first neutralizing Nb against poxvirus by encompassing a comprehensive analysis of epitope specificity, the mechanism of action, and its protective efficacy in mouse models. This work presents a potential candidate for the prophylaxis and therapy of orthopoxvirus infections. Moreover, the findings also provide critical insights that could inform the development of novel and effective vaccines.

## Materials and methods

### Cell lines and viruses

BHK-21 (ATCC, CCL-10) and Vero-E6 cells (ATCC, CRL-1586) were cultured in Dulbecco’s Modified Eagle’s Medium (DMEM; Gibco, New York, NY, USA), supplemented with 10% fetal bovine serum (FBS; Thermo Fisher Scientific). The African green monkey kidney cell line BS-C-1 (ATCC, CCL-26) was maintained at 37 °C in minimum essential medium (MEM; Thermo Fisher) enriched with 10% FBS. Expi293F cells and expi293 expression medium were acquired from Thermo Fisher (Waltham, MA, USA).

HeLa cells were used for the propagation of vaccinia virus strain Western Reserve (VACV-WR, ATCC VR-119) and Modified Vaccinia virus Ankara (MVA) as previously described; the EEV forms were obtained from the medium and IMV forms were purified from lytic cells. The viral titers of the purified virus stocks were determined through a plaque assay employing BS-C-1 cells. All experiments involving live vaccinia virus were conducted adhering to Biosafety Level 2 (BSL-2) guidelines. Mpox virus (MPXV, strain IIb.c.1) propagation and tittering were performed in monolayer cultures of Vero cells. Experiments involving infectious MPXV were executed under stringent BSL-3 conditions at the Institute of Laboratory Animal Science, Chinese Academy of Medical Sciences, and Peking Union Medical College, Beijing, China, by personnel with specific training in handling high-contagious pathogens.

### Expression and purification of recombinant proteins

To express His-tagged recombinant MPXV proteins, plasmids were engineered utilizing established molecular biology techniques. For A29L, M1R, and E8L, upon successful transformation into *E. coli*, the bacterial cultures were induced, and the resulting bacterial pellets were subsequently resuspended in HBS buffer (10 mM HEPES, 150 mM NaCl) supplemented with 1 mM phenylmethylsulfonylfluoride. This mixture was then subjected to ultrasonication to disrupt the cells, followed by purification. The purification process involved the use of Ni-NTA resin to specifically bind the His-tagged proteins. In addition, plasmids expressing H3L, A35R, or B6R were transfected into 293F cells by FectroPRO^®^; after 5 days of expression, proteins were purified by Ni-NTA resin. Subsequently, gel filtration chromatography was employed using a Superdex™ 75 Increase 10/300 GL column to separate the proteins based on their size. This comprehensive purification strategy was executed to obtain highly pure His-tagged recombinant MPXV proteins for Nb screening.

### Analysis of membrane protein conservation

To explore the structures of the extracellular regions of six membrane proteins from VACV and MPXV, corresponding sequence data were retrieved from the UniProt database. Subsequently, AlphaFold2, a state-of-the-art protein structure prediction tool, was employed to generate the three-dimensional structures of these proteins. The models with the highest prediction scores were selected for further analysis. Thereafter, PyMOL was utilized to compare the predicted structures from both viral species. This comparative analysis aims to provide insights into the structural similarities and sequence differences between these proteins.

### Nb selection by phage display

The selection of Nbs through phage display technology was conducted in our laboratory as previously described^24,25^. A fully synthetic library of Nb phages with high diversity was developed. The selection process involved both immunotube and bead-conjugated recombinant antigen panning, following established protocols^33^. Four rounds of panning were executed. In the first and third panning rounds, MPXV membrane proteins were biotinylated using EZ-Link™ Sulfo-NHS-LC-Biotin (Thermo Fisher) and then isolated using streptavidin-coated magnetic Dynabeads™ M-280 (Thermo Fisher). For the second and fourth rounds, the purified membrane proteins were adsorbed onto Nunc MaxiSorp immunotubes (Thermo Fisher) overnight at 4 °C. During each panning round, ~10^11^ phage particles were incubated with antigen-coated immunotubes or biotinylated antigens for 1 h. Following the binding reaction, unbound phages were removed through repeated washing with PBS, while the bound phages were eluted using Glycine-HCl (pH 3.0) and neutralized with Tris-Cl (pH 9.0). Phages from the previous round were used to infect the next round. After four rounds of screening, phage ELISA was performed using approximately 500 individual colonies per antigen. This was done using anti-CM13 antibody (Sino Biological, Beijing, China) in plates coated with recombinant antigens. Positive clones were then submitted for antibody sequencing, and unique sequences were cloned for Nb expression.

### Expression and purification of Nbs

The Nbs were produced and purified according to a previously established methodology^33^. In brief, the gene encoding the Nb was inserted into the pET-22b expression vector, and *E. coli* BL21(DE3) was employed for protein expression. To generate bivalent and trivalent Nbs, a (GGGGS)_3_ linker was introduced between the two monomers of the Nb. The expressed proteins were fused to a 6*×* His tag at the C-terminus and subsequently purified utilizing Ni Sepharose 6 Fast Flow column chromatography (GE Healthcare, Boston, MA, USA). The purified proteins were eluted using a gradient of imidazole, typically at 300 mM. The identity and purity of the proteins were confirmed by SDS-PAGE, followed by Coomassie Brilliant Blue staining. These purified Nbs are ready to be used for further characterization and application in neutralization and protection assays.

### Production and purification of Fc-fusion Nbs

The Nb sequence was subcloned into a panel of plasmids engineered for the production of Fc-fusion proteins. These plasmids were designed to harbor the hEF1-HTLV promoter and to encode an interleukin-2 signal peptide (IL-2 ss) at the N-terminus and the Fc-region of either human or mouse IgG at the C-terminus. Maxiprep-purified plasmids were introduced into Expi293F cells (Thermo Fisher) via FectoPRO^®^ reagent-mediated transfection, with the aid of Fectro Booster (Polyplus Transfection, Illkirch-Graffenstaden, France) to enhance expression. Following a 5-day suspension culture, the cell supernatants were harvested, and the Fc-fusion proteins were isolated using Protein A MagBeads (GenScript, Nanjing, China). Protein A binding was followed by elution with Glycine-Cl (pH 3.5) and neutralization with Tris-Cl (pH 9.0). The elution buffer containing glycine was subsequently exchanged for phosphate-buffered saline (PBS, pH 7.0). The purity and identity of the proteins were confirmed by SDS-PAGE and western blotting assay, with standard protocols. Additionally, the murine antibody 7D11^19^ was synthesized based on sequences available in the literature and successfully expressed using 293F cells.

### Affinity measurement and competition-binding assay

SPR was employed to assess the affinity between recombinant MPXV membrane proteins and Nbs using a BiaCore T200 system. The proteins were immobilized on CM5 sensor chips (GE Healthcare) following a standard procedure. The sample and reference flow channels’ surfaces were activated with a 1:1 mixture of 0.1 M *N*-hydroxysuccinimide (NHS) and 3-*N*, *N*-dimethylaminopropyl-*N*-ethycarbodiimide (EDC) at a flow rate of 10 µL/min. Post activation, all surfaces were blocked with 1 M ethanolamine (pH 8.0). The running buffer used was PBS-P (phosphate-buffered saline, calcium- and magnesium-free, pH 7.0, with 0.05% P20). Approximately 400 response units of antigens were immobilized on the sensor chips for affinity assays. Serial dilutions of Nbs were used as the ligands and were passed over the chip surface. After each cycle of association and dissociation, the sensor surface was regenerated with glycine-HCl (pH 2.5) to remove any unbound material. The affinity data were collected and analyzed using BIAevaluation software, which fit the data to a 1:1 binding model.

For the competition-binding assay, M1R-01 and 7D11 antibodies were diluted to specific concentrations. Both M1R-01 and 7D11, as well as IgG-iso antibodies, were first passed over the sensor surface for 500 s. Subsequently, the other antibody was passed over the surface without dissociation for an additional 500 s. The binding curves obtained were then analyzed using BIAevaluation software to interpret the results and address the research questions.

### Neutralization assays

The neutralizing capacity of antibodies was evaluated using a plaque reduction neutralization test (PRNT) assay against vaccinia virus strains western reserve (VACV-WR), modified Ankara (MVA), or monkeypox virus (MPXV). For Vaccinia virus strains WR and MVA, the PRNT assay was conducted on BS-C-1 cells, while for MPXV, the neutralization assay was performed on Vero-E6 cells in a certificated Biosafety Level 3 (BSL-3) laboratory. The antibodies were serially diluted with PBS, and these dilutions were combined with 50–100 plaque-forming units (PFU) of virus EEV or IMV, which were then incubated at 37 °C for 1 h, for EEV, 10 μg/mL 7D11^19^ was added to block IMV during the experiment. Subsequently, a monolayer of BS-C-1 or Vero cells was infected with the antibody/virus mixture, and the mixture was allowed to incubate for 2 h at 37 °C. After that, the mixture was removed, and the cells were overlaid with semisolid Earle’s basal minimum essential medium (EMEM) supplemented with 2.5% FBS, 1.5% methylcellulose, and 100 U/mL penicillin-streptomycin. The plates were then incubated at 37 °C under a 5% CO_2_ atmosphere for 48 h. Two days post-infection, the cells were fixed with 4% paraformaldehyde (Solarbio, Beijing, China) for 30 min at room temperature before being stained with a 0.1% crystal violet solution. The plaques were then enumerated. The percentage of neutralization was calculated relative to the plaque number in the absence of an antibody (PBS control group).

### Mouse protection by intranasal administration of Tri-M1R-01

In the protective efficacy study of the trivalent Nb Tri-M1R-01, 7–8-week-old female BALB/c mice were utilized. Following isoflurane anesthesia, a dose of 10 mg/kg antibody (30 μL volume) was administered intranasally to the mice. A control group received an equivalent dose of the unrelated control Nb (NB10) via the same route. The mice rapidly inhaled the liquid. 30 min post-administration, 0.5 *×* 10^5^ PFU of VACV-WR (10 μL volume) were instilled into the noses of the mice, after which they were returned to their cages. The weight of the mice was recorded daily. A body weight loss exceeding 20% of the original weight was considered lethal. On day 5 post-challenge, blood, spleen, and lung samples were collected from approximately 4 to 5 mice per group. Blood DNA samples were utilized for quantitative reverse transcription PCR (qRT-PCR) to detect VACV using the following primers^34^, forward primer: 5′-CATCATCTGGAATTGTCACTACTAAA-3′; reverse primer: 5′-ACGGCCGACAATATAATTAATGC-3′. The spleen weight was recorded and used to calculate the spleen-to-body weight ratio (mg/g). For the lung tissue, PBS was added at a proportion of 0.1 g/mL to prepare tissue homogenates. These homogenates were diluted in various gradients and subsequently inoculated onto BS-C-1 monolayer cells to calculate the virus plaque growth. This experimental protocol was approved by the Beijing Experimental Animal Research Center (BLARC-SSYY-DW/013-JL/001) to ensure adherence to ethical guidelines and the humane treatment of experimental animals.

### In vivo protection by intraperitoneal injection of Fc-M1R-01

Mice were intraperitoneally administered with either Fc-M1R-01 or an irrelevant control antibody (anti-GP73) at a dose of 10 mg/kg. Following this, the mice were intranasally infected with 0.5 *×* 10^5^ PFU of VACV-WR. On day 2 post-infection, the mice received an additional 10 mg/kg dose of the same antibodies via the same route. Mice were weighed daily to monitor disease progression. On day 5, half of the mice in each group were sacrificed, and blood, spleen, and lung samples were collected for further analysis. The detection process, including qRT-PCR for viral load detection and assessment of spleen and lung tissue, was conducted as described above.

### Crystallization of the complex and data collection

The purified M1R protein was combined with the M1R-01 at a 1:1 molar ratio to form a protein–Nb complex. Crystals of this complex were successfully grown at 16 °C in sitting drops, which were positioned over wells containing a solution consisting of 0.1 M ammonium acetate, 0.1 M BIS-Tris at pH 5.5, and 17% w/v polyethylene glycol (PEG) 10,000. The process involved the addition of 150 nL of the M1R–Nb complex in the HBS buffer to 150 nL of well solution. Subsequently, the crystals were harvested and soaked in a cryoprotective solution containing 0.1 M ammonium acetate, 0.1 M BIS-Tris at pH 5.5, 17% w/v PEG 10,000, and 20% glycerol. Following the soaking process, the crystals were rapidly frozen in liquid nitrogen to maintain their structure. Diffraction data were collected at 100 K and at a wavelength of 0.987 Å using the BL18U1 beamline at the Shanghai Synchrotron Radiation Facility. The diffraction data were then processed using the HKL2000 software^35^. A comprehensive summary of the data-processing statistics is provided in Supplementary Table S3.

### Structure determination and refinement

The structural determination of the M1R–Nb complex utilized the molecular replacement method, implemented through PHASER within the CCP4 software suite^36^. The search models incorporated into the process included the Nb M1R-01, which was predicted by AlphaFold2 (PMID: 34265844), and L1R from the 4U6H structure. Model construction and refinement were then carried out using COOT and PHENIX, respectively^37,38^. The final Ramachandran plot statistics revealed that 92.89% of residues were in the favored region, 6.37% were in the allowed region, and 0.74% were classified as outliers. Detailed statistics regarding the refinement process are provided in Supplementary Table S3. Structural visualization was performed using Chimera X^39^.

### Pull-down assays

The M1R point mutants, each bearing a C-terminal Flag-tag, were engineered using the KOD-Plus-Mutagenesis Kit (TOYOBO, Japan) in accordance with the manufacturer’s standard protocol. Subsequently, the maxiprep-purified plasmids were transfected into 293T cells, and the cells were allowed to grow for 36 h. Following this, the cells were washed three times with PBS and lysed using IP lysis buffer (Thermo Fisher) that was supplemented with a cocktail of protease inhibitors to maintain protein integrity. Pierce® Protein A/G Magnetic beads (Thermo Fisher) were pre-incubated with mFc-M1R-01 for 2 h at room temperature to facilitate the subsequent binding process. The cell lysate was then centrifuged at 14,000*× g* for 10 min at 4 °C to pellet the cellular debris. A volume of 100 μL of the supernatant was retained as the input control group by adding it to the loading buffer for subsequent analysis. The remaining supernatant was then added to the magnetic beads for overnight incubation at 4 °C to allow for binding. The following day, the magnetic beads were captured using a magnetic holder, and the supernatant was discarded. The protein-bound beads were then subjected to five washes with IP washing buffer (0.05 M Tris, 0.15 M NaCl, 0.1% Tween-20, pH 7.0) to remove any unbound material. Subsequently, the protein was eluted using Glycine-Cl (pH 3.5), and the sample was immediately neutralized with Tris-Cl (pH 9.0). After the addition of the loading buffer, western blot analysis was conducted to detect the mutant proteins. For the western blot detection, the primary antibody used was anti-Flag (Abmart, Shanghai, China), and the secondary antibody was anti-mouse-HPR (Jackson, USA).

### Co-IP assays

The mutant variants of the mFc-M1R-01 were generated using a protocol similar to that employed for MIR, with the subsequent co-transfection of these mutant plasmids with the M1R-Flag construct into 293T cells. Following a 48-h expression period, the cells were washed three times with pre-chilled PBS and then lysed on ice for 30 min in an IP lysis buffer that was supplemented with a protease inhibitor cocktail to maintain protein stability. Following lysis, the cell lysates were centrifuged to pellet cellular debris. The cleared lysates were then incubated overnight with protein A/G magnetic beads to facilitate the capture of the mutant proteins. After incubation, the beads were subjected to five washes with IP washing buffer to remove any non-specifically bound material. Subsequently, the bound proteins were eluted using Glycine-Cl (pH 3.5), and the sample was immediately neutralized with Tris-Cl (pH 9.0). The eluted proteins were then analyzed by western blotting assay, which were conducted as described above.

### Investigation of the temporal dynamics of antibody neutralization

To assess virus–cell binding, pre-cooled Hela cells were incubated with VACV containing 50 μg/mL of Tri-M1R-01 or an irrelevant Nb (NB10, which targets coronaviruses) at 4 °C for 1 h to facilitate virus attachment. The cells were then harvested and washed twice with pre-cooled PBS to remove unbound virus. Total cellular DNA was extracted using a commercial cell DNA extraction kit (TIANGEN, China). A quantitative polymerase chain reaction (qPCR) was performed using standard procedures. The reaction conditions included an initial denaturation step at 50 °C for 2 min and 95 °C for 2 min, followed by 45 cycles of amplification with denaturation at 95 °C for 10 s and annealing/extension at 60 °C for 40 s. For the post-binding assay, a monolayer of BS-C-1 cells in 48-well plates was incubated with approximately 50 PFU of VACV at 4 °C for 1 h. The cells were then washed with PBS, and this time point was designated as 0 h. MEM containing 2.5% FBS, 1.5% methylcellulose, and either 50 μg/mL Tri-M1R-01 or NB10 was added at timepoints of 0, 2-, 4-, 6-, and 8-h post-infection. After an additional 40-h incubation at 37 °C, the plates were stained with a solution of 0.1% crystal violet, and plaques were enumerated to quantify the remaining infectious virus.

### Quantification and statistical analysis

Figures and statistical analyses were conducted using GraphPad Prism 8. Statistical significance between groups was assessed using either a *t*-test or one-way ANOVA, as indicated in the respective figure legends.

## Supplementary information

Supplementary Information

## Data Availability

We have deposited the structure of M1R and Nb complex in the Protein Data Bank (PDB) with the accession code 9J7Y (https://www.rcsb.org/).
