# Supplementary material for: Identification of neutralizing nanobodies protecting against poxvirus infection
Source: Cell Discov. 2025 Mar 25;11:31. doi: 10.1038/s41421-025-00771-7 (PMC11937253; doi:10.1038/s41421-025-00771-7)
Supplement: Supplementary file 1 — Supplementary Information [file 41421_2025_771_MOESM1_ESM.pdf]

Supplementary Materials

Identification of neutralizing nanobodies protecting against poxvirus infection

Xuehua Yang[1, 2, #], Li Guo[1, 4, #], Huarui Duan[1, #], Miao Fan[1, #], Fengwen Xu[1], Xiaojing Chi[1, 2], Shengnan Pan[1], Xiuying Liu[1, 2], Xinhui Zhang[1], Peixiang Gao[1], Fangyuan Zhang[1], Xinyi Wang[1], Fei Guo, Jiwan Ge[*, 1], Lili Ren[*, 1, 4], Wei Yang[*, 1, 2, 3]

[1] Key Laboratory of Pathogen Infection Prevention and Control (Ministry of Education), National Institute of Pathogen Biology, Chinese Academy of Medical Sciences & Peking Union Medical College, Beijing, China.

[2] NHC Key Laboratory of Systems Biology of Pathogens, National Institute of Pathogen Biology, Chinese Academy of Medical Sciences & Peking Union Medical College, Beijing, China.

[3] State Key Laboratory of Respiratory Health and Multimorbidity, Chinese Academy of Medical Sciences & Peking Union Medical College, Beijing, China.

[4] NHC Key Laboratory of System Biology of Pathogens and Christophe Mérieux Laboratory, National Institute of Pathogen Biology, Chinese Academy of Medical Sciences & Peking Union Medical College, Beijing, China.

* Corresponding author:

Wei Yang: wyang@ipb.pumc.edu.cn

Lili Ren: renliliipb@163.com

Jiwan Ge: gejiwan@ipbcams.ac.cn

# These authors contributed equally.

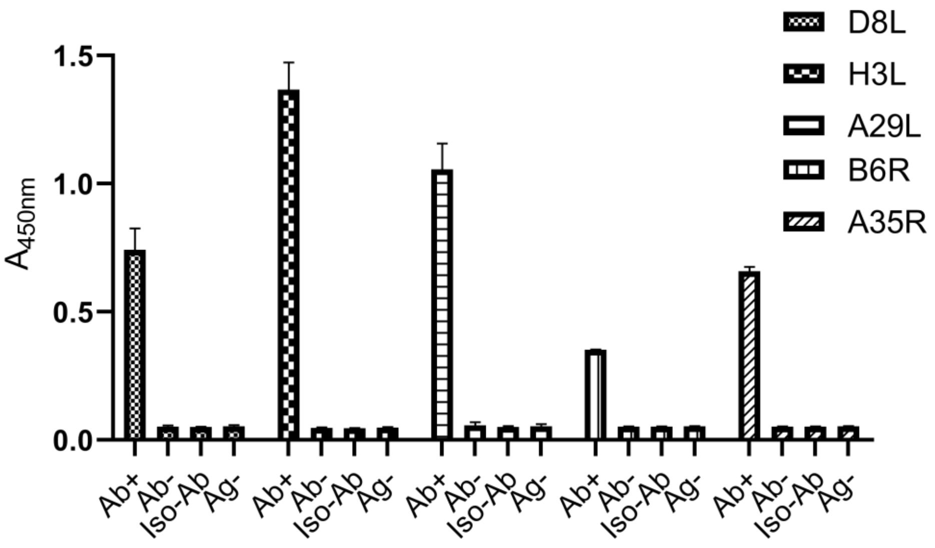

Supplementary Fig. S1. Detection of purified membrane proteins by ELISA using commercial antibodies. Purified membrane proteins were immobilized onto an Enzyme-Linked Immunosorbent Assay plate at a concentration of 2 μg/mL. Subsequently, commercial antibodies were applied to identify the target antigens. Isotype-matched IgGs served as a negative control to ensure specificity of the antibody binding.

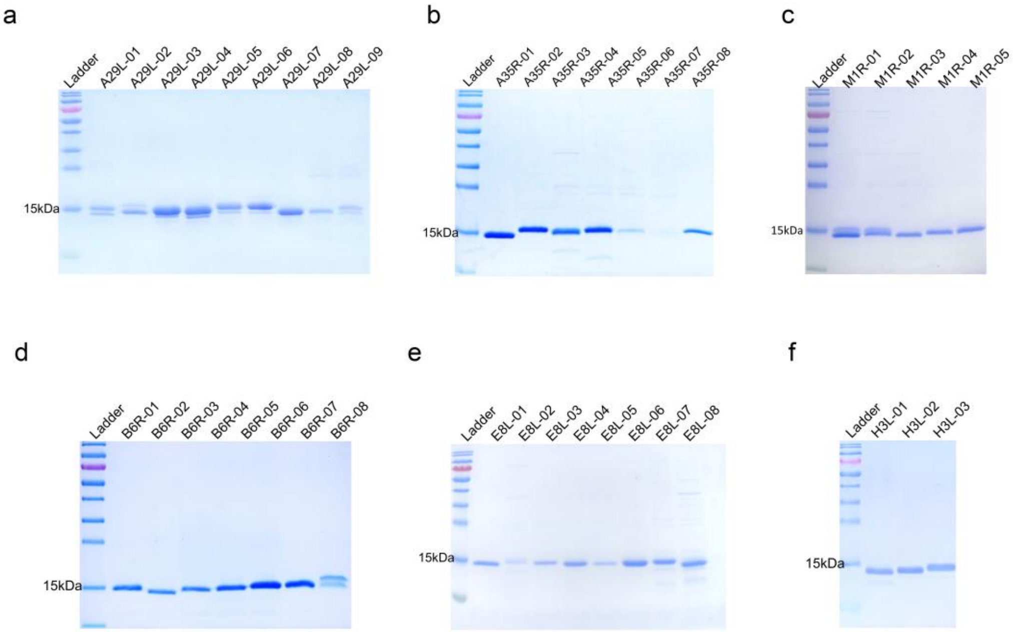

Supplementary Fig. S2. SDS-PAGE of all purified Nbs. Six groups of Nbs were analyzed by SDS-PAGE and Coomassie blue staining, 15 kDa marker are labeled. **a-f** Nbs targeting MPXV A29L (a), A35R (b), M1R (c), B6R (d), E8L (e) and H3L (f).

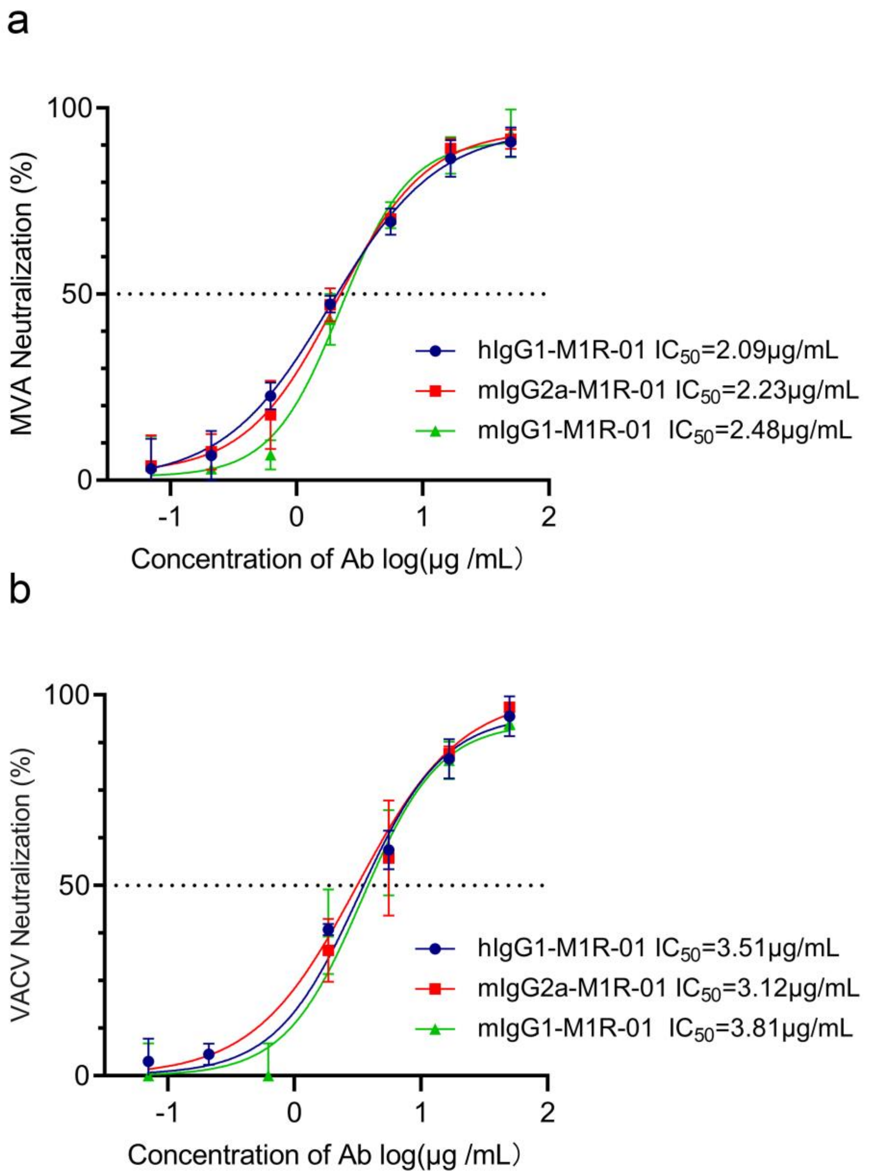

Supplementary Fig. S3. Neutralizing activity of IgG-M1R-01 against VACV. a, b Three types of IgG fused M1R-01 against MVA (a) and VACV-WR (b). Data represent one of two independent experiments, shown as mean ± SD of assay triplicates.

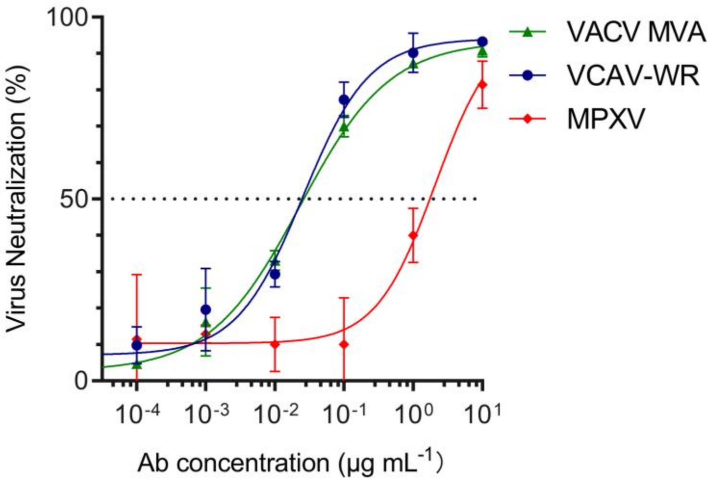

Supplementary Fig. S4. Neutralizing activity of 7D11 against VACV and MPXV. Neutralizing activity of 7D11 against MVA (green), VACV-WR (blue) and MPXV (red) tested by PRNT. Data represent one of two independent experiments, shown as mean ± SD of assay triplicates.

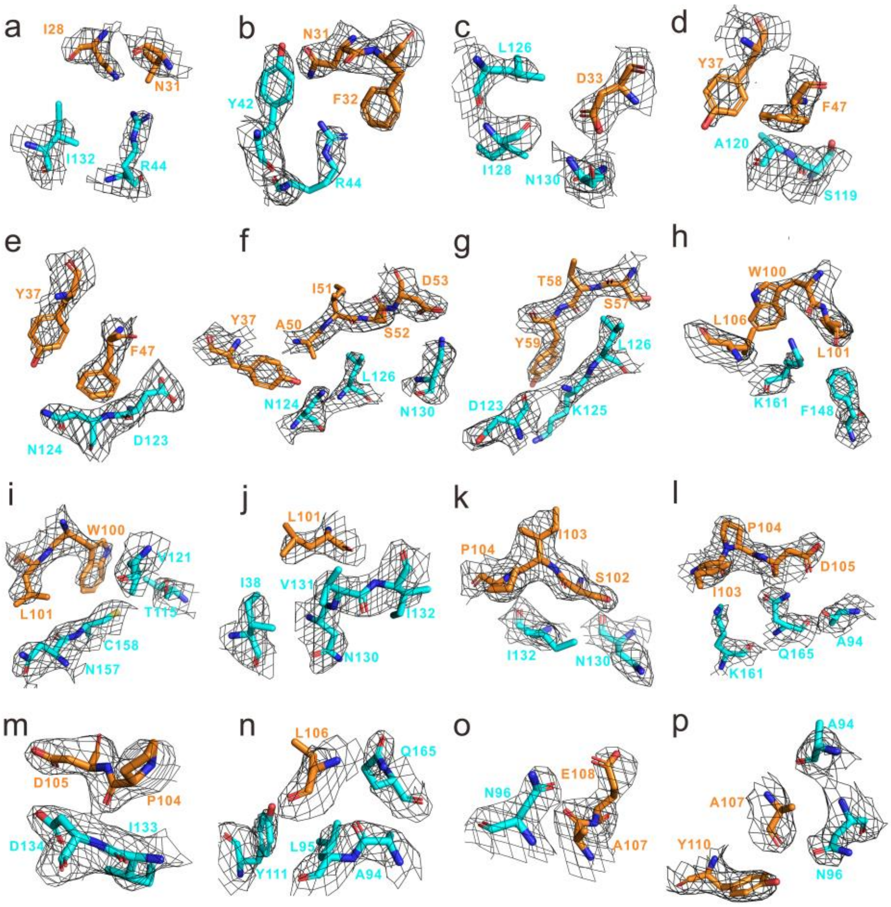

Supplementary Fig. S5. The $2F_o - F_c$ electron density maps contoured at 1.5σ at the binding interface between the nanobody M1R-01 (tv-orange) and M1R (cyan).

Supplementary Table S1. Sequences of nanobodies selected by phage-display.

Target	Nb	sequence
E8L	E8L-01	QVQLVESGGGLVQAGGSLRLSCAASGRIFINRVMGWYRQAPGKEREFVAGIAQGGSTNYADSVKGRFTISRDNAKNTVYLQMNSLKPEDTAVYYCAAKFWNWHHDHRGYDYWGQGTQVTVSS
	E8L-02	QVQLVESGGGLVQAGGSLRLSCAASGRIFTDSYMGWYRQAPGKEREFVACITTGSSTYYADSVKGRFTISRDNAKNTVYLQMNSLKPEDTAVYYCAARFRYTVQHWFHTKFDYWGQGTQVTVSS
	E8L-03	QVQLVESGGGLVQAGGSLRLSCAASGFIFTTRGMGWYRQAPGKEREFVACIFPGSSTNYADSVKGRFTISRDNAKNTVYLQMNSLKPEDTAVYYCAAEGPYRGFNFKKNLYDYWGQGTQVTVSS
	E8L-04	QVQLVESGGGLVQAGGSLRLSCAASGRIFGRRAMGWYRQAPGKEREFVAAISNGFNTNYADSVKGRFTISRDNAKNTVYLQMNSLKPEDTAVYYCAATVSRRGEQTFFYDYWGQGTQVTVSS
	E8L-05	QVQLVESGGGLVQAGGSLRLSCAASGRIFDTVEMGWYRQAPGKEREFVASISGGDSTYYADSVKGRFTISRDNAKNTVYLQMNSLKPEDTAVYYCAARSARNRWSIIFYDYWGQGTQVTVSS
	E8L-06	QVQLVESGGGLVQAGGSLRLSCAASGFIFTSTRMGWYRQAPGKEREFVAGISIGGNTNYADSVKGRFTISRDNAKNTVYLQMNSLKPEDTAVYYCAARYYYFHNHNFAYDYWGQGTQVTVSS
	E8L-07	QVQLVESGGGLVQAGGSLRLSCAASGSIFKRYKMGWYRQAPGKEREFVACINKGGDTNYADSVKGRFTISRDNAKNTVYLQMNSLKPEDTAVYYCAVKRHAKAYGEFFYDYWGQGTQVTVSS
	E8L-08	QVQLVESGGGLVQAGGSLRLSCAASGFIFTNWGMGWYRQAPGKERELVAAITSGSSTNYADSVKGRFTISRDNAKNTVYLQMNSLKPEDTAVYYCAAIRGFSLIYQFFAQYDYWGQGTQVTVSS
H3L	H3L-01	QVQLVESGGGLVQAGGSLRLSCAASGRIFWWVPMGWYRQAPGKEREFVAGISKGDSTYYADSVKGRFTISRNNAKNTVYLQMNSLKPEDTAVYYCAANPRNNRFRAGVYDYWGQGTQVTVSS
	H3L-02	QVQLVESGGGLVQAGGSLRLSCAASGRIFLHRIMGWYRQAPGKERELVASISRGDNTYYADSVKGRFTISRDNAKNTVYLQMNSLKPEDTAVYYCAAGTGRHKDDHKDYDYWGQGTQVTVSS
	H3L-03	QVQLVESGGGLVQAGGSLRLSCAASGRIFFFAKMGWYRQAPGKEREFVASIVYGDITYYADSVKGRFTISRDNAKNTVYLQMNSLKPEDTAVYYCAAKPHWPSRRPRRYDYWGQGTQVTVSS
A29L	A29L-01	QVQLVESGGGLVQAGGSLRLSCAASGRIFWAGLMGWYRQAPGKEREFVAGINLGGSTYYADSVKGRFTISRDNAKNTVYLQMNSLKPEDTAVYYCAAQVHVVRFQIQPYDYWGQGTQVTVSS
	A29L-02	QVQLVESGGGLVQAGGSLRLSCAASGFIFPLFPMGWYRQAPGKEREFVAGISWGGDTYYADSVKGRFTISRDNAKNTVYLQMNSLKPEDTAVYYCAVQFRIEKYYGKRYDYWGQGTQVTVSS
	A29L-03	QVQLVESGGGLVQAGGSLRLSCAASGRIFLLDDMGWYRQAPGKEREFVASI

		TWGSSTYYADSVKGRFTISRDNAKNTVYLQMNSLKPEDTAVYYCAVIQYFFWEYSTTLDYWGQGTQVTVSS
	A29L-04	QVQLVESGGGLVQAGGSLRLSCAASGFILSDVNMGWYRQAPGKEREFVAGITHGGSTYYADSVKGRFTISRDNAKNTVYLQMNSLKPEDTAVYYCAAHALINKHYSAVYDYWGQGTQVTVSS
	A29L-05	QVQLVESGGGLVQAGGSLRLSCAASGRIFNRNGMGWYRQAPGKEREFVAGITGGASTYYADSVKGRFTISRDNAKNTVYLQMNSLKPEDTAVYYCAVRGILDLEFFKALDYWGQGTQVTVSS
	A29L-06	QVQLVESGGGLVQAGGSLRLSCAASGRIFTVNAMGWYRQAPGKEREFVAGITAGGNTYYADSVKGRFTISRDNAKNTVYLQMNSLKPEDTAVYYCAVHNLIEHGKRDFFDYWGQGTQVTVSS
	A29L-07	QVQLVESGGGLVQAGGSLRLSCAASGRIFKWASMGWYRQAPGKEREFVAGIINGGNTYYADSVKGRFTISRDNAKNTVYLQMNSLKPEDTAVYYCAVTGFQARLDVLFYDYWGQGTQVTVSS
	A29L-08	QVQLVESGGGLVQAGESLRLSCAASGFIFDLVDMGWYRQAPGKEREFVAAITWGGSTNYADSVKGRFTISRDNAKNTVYLQMNSLKPEDTAVYYCAATWQDIDWNEWITSQRYDYWGQGTQVTVSS
	A29L-09	QVQLVESGGGLVQAGGSLRLSCAASGRIFHVHGMGWYRQAPGKEREFVAGITHGRRTYYADSVKGRFTISRDNAKNTVYLQMNSLKPEDTAVYYCAALQVHNPRQGTFYDYWGQGTQVTVSS
**B6R**	B6R-01	QVQLVESGGGLVQAGGSLRLSCAASGFISNRREMGWYRQAPGKEREFVAAISTGRSTYYADSVKGRFTISRDNAKNTVYLQMNSLKPEDTAVYYCAANDLHRNKGHAIYDYWGQGTQVTVSS
	B6R-02	QVQLVESGGGLVQAGGSLRLSCAASGRIFRRNVMGWYRQAPGKEREFVAAITKGGYTNYADSVKGRFTISRDNAKNTVYLQMNSLKPEDTAVYYCAARYFHRKRHKWRHDYWGQGTQVTVSS
	B6R-03	QVQLVESGGGLVQAGGSLRLSCAASGRILHRKGMGWYRQAPGKEREFVAAINKGGSTNYADSVKGRFTISRDNAKNTVYLQMNSLKPEDTAVYYCAVGKKRRTWINRHYDYWGQGTQVTVSS
	B6R-04	QVQLVESGGGLVQAGGSLRLSCAASGRIFASDRMGWYRQAPGKEREFVAAISYGSRTNYADSVKGRFTISRDNAKNTVYLQMNSLKPEDTAVYYCAVWAFHRSWKERSHWHDYWGQGTQVTVSS
	B6R-05	QVQLVESGGGLVQAGGSLRLSCAASGRIFHPGAMGWYRQAPGKEREFVAGISSGGNTYYADSVKGRFTISRDNAKNTVYLQMNSLKPEDTAVYYCAAPFRWVKATGRFLDYWGQGTQVTVSS
	B6R-06	QVQLVESGGGLVQAGGSLRLSCAASGRIFRKRNMGWYRQAPGKEREFVASISRGSNTYYADSVKGRFTISRDNAKNTVYLQMNSLKPEDTAVYYCAVWRHRTFVRWKHFDYWGQGTQVTVSS
	B6R-07	QVQLVESGGGLVQAGKFTLIVCGIWFYFPSVTMGWYRQAPGKERELVAAITHGSTTYYADSVKGRFTISRDNAKNTVYLQMNSLKPEDTAVYYCAVKHFFGKDWPWVYDYWGQGTQVTVSS
	B6R-08	QVQLVESGGGLVQAGGSLRLSCAASGFISNRREMGWYRQAPGKEREFVAAISTGRSTYYADSVKGRFTISRDNAKNTVYLQMNSLKPEDTAVYYCAANDLHRNKGHAIYDYWGQGTQVTVSS

**A35R**	A35R-01	QVQLVESGGGLVQAGGSLRLSCAASGSIFVLNYMGWYRQAPGKEREFVASISRGRRTYYADSVKGRFTISRDNAKNTVYLQMNSLKPEDTAVYYCAAYIRRPHYRWYDYWGQGTQVTVSS
	A35R-02	QVQLVESGGGLVQAGGSLRLSCAASGSIFHHGKMGWYRQAPGKEREFVAAISRGGSTYYADSVKGRFTISRDNAKNTVYLQMNSLKPEDTAVYYCAAWVIRKHYPYHWYDYWGQGTQVTVSS
	A35R-03	QVQLVESGGGLVQAGGSLRLSCAASGRIFYYRDMGWYRQAPGKEREFVAAISKGRSTNYADSVKGRFTISRDNAKNTVYLQMNSLKPEDTAVYYCAAWGIRRHHWRHWYDYWGQGTQVTVSS
	A35R-04	QVQLVESGGGLVQAGGSLRLSCAASGRIFRFTKMGWYRQAPGKEREFVAAITDGDHTYYADSVKGRFTISRDNAKNTVYLQMNSLKPEDTAVYYCAAWYRKRRHWYWLYDYWGQGTQVTVSS
	A35R-05	QVQLVESGGGLVQAGGSLRLSCAASGRIFPIRHMGWYRQAPGKEREFVASIAAGGSTYYADSVKGRFTISRDNAKNTVYLQMNSLKPEDTAVYYCAAYIRRPHYRWYDYWGQGTQVTVSS
	A35R-06	QVQLVESGGGLVQAGGSLRLSCAASGRIFSHRPMGWYRQAPGKEREFVASISRGGNTYYADSVKGRFTISRDNAKNTVYLQMNSLKPEDTAVYYCAARQWRHFGHSHRHDYWGQGTQVTVSS
	A35R-07	QVQLVESGGGLVQAGGSLRLSCAASGRIFWRKYMGWYRQAPGKEREFVAGIARGRTTNYADSVKGRFTISRDNAKNTVYLQMNSLKPEDTAVYYCAAEFSVRPWHPYRYDYWGQGTQVTVSS
	A35R-08	QVQLVESGGGLVQAGGSLRLSCAASGRILRARSMGWYRQAPGKEREFVAAIAKGGITNYADSVKGRFTISRDNAKNTVYLQMNSLKPEDTAVYYCAVIWTKTRWWHWLPQFTYDYWGQGTQVTVSS
**M1R**	M1R-01	QVQLVESGGGLVQAGGSLRLSCAASGFIFHNFDMGWYRQAPGKEREFVAAISDNGRSTYYADSVKGRFTISRDNAKNTVYLQMNSLKPEDTAVYYCAVAWLSIPDLAEWYDYWGQGTQVTVSS
	M1R-02	QVQLVESGGGLVQAGGSLRLSCAASGFIFDHWEMGWYRQAPGKEREFVASISAGLSTNYADSVKGRFTISRDNAKNTVYLQMNSLKPEDTAVYYCAARHFTAVFWKNSYDYWGQGTQVTVSS
	M1R-03	QVQLVESGGGLVQAGGSLRLSCAASGSIFETGAMGWYRQAPGKEREFVAAITGWDTNYADSVKGRFTISRDNAKNTVYLQMNSLKPEDTAVYYCAASHWISFYWEHYHPYDYWGQGTQVTVSS
	M1R-04	QVQLVESGGGLVQAGGSLRLSCAASGRIFTARYMGWYRQAPGKEREFVAAIAAGDTTNYADSVKGRFTISRDNAKNTVYLQMNSLKPEDTAVYYCAAWWAWSPVNRLHYDYWGQGTQVTVSS
	M1R-05	QVQLVESGGGLVQAGGSLRLSCAASGFIFYAWHMGWYRQAPGKERELVASIAYGGDTNYADSVKGRFTISRDNAKNTVYLQMNSLKPEDTAVYYCAAVHFTHHKWDQEYDYWGQGTQVTVSS

Supplementary Table S2. The binding affinity of the Nbs to their target proteins was characterized using Surface Plasmon Resonance (SPR).

Target	Nb	k_{on} ($M^{-1}s^{-1}$)	k_{off} (s^{-1})	K_D (nM)
**E8L**	E8L-01	4.35×10^6	0.0029	0.66
	E8L-02	1.84×10^5	0.0003	1.69
	E8L-03	6.62×10^5	0.0034	5.81
	E8L-04	8.76×10^5	0.0015	1.77
	E8L-05	8.38×10^5	0.0020	2.39
	E8L-06	9.10×10^5	0.0105	11.55
	E8L-07	5.39×10^5	0.0018	3.17
	E8L-08	6.46×10^5	0.0020	3.11
**H3L**	H3L-01	1.87×10^6	0.0209	11.15
	H3L-02	7.65×10^5	0.0725	94.79
	H3L-03	2.61×10^5	0.0092	35.16
**A29L**	A29L-01	3.71×10^5	0.0077	20.82
	A29L-02	1.13×10^6	0.0218	19.27
	A29L-03	1.04×10^6	0.0036	3.47
	A29L-04	1.27×10^5	0.0023	17.99
	A29L-05	4.61×10^5	0.0065	14.13
	A29L-06	2.20×10^5	0.0027	12.04
	A29L-07	5.35×10^5	0.0045	8.36
	A29L-08	1.71×10^5	0.0054	31.49
	A29L-09	4.18×10^5	0.0389	9.32
**B6R**	B6R-01	1.28×10^6	0.0522	40.82
	B6R-02	2.09×10^2	0.0029	14000
	B6R-03	1.10×10^5	0.3495	3182
	B6R-04	3.99×10^4	0.0412	1032
	B6R-05	8.9	0.0008	95420
	B6R-06	4.62×10^5	0.0056	12.1
	B6R-07	NA	NA	NA
	B6R-08	NA	NA	NA
**A35R**	A35R-01	9.331	0.0011	119300
	A35R-02	9.44×10^4	0.0028	29.47
	A35R-03	NA	NA	NA
	A35R-04	6.77×10^6	0.0077	1.13
	A35R-05	NA	NA	NA
	A35R-06	NA	NA	NA
	A35R-07	NA	NA	NA
	A35R-08	NA	NA	NA

	M1R-01	4.76×10^4	0.0018	37.67
	M1R-02	6.83×10^4	0.0016	23.49
**M1R**	M1R-03	1.61×10^6	0.0401	24.89
	M1R-04	NA	NA	NA
	M1R-05	4.28×10^4	0.0037	85.91

*The association rate constant (K_{on}), dissociation rate constant (K_{off}) and K_D are indicated for each Nb. Nbs with affinities deemed too weak to yield reliable data are indicated as "NA."

Supplementary Table S3. Data collection and refinement statistics.

	M1R-M1R-01 complex
**Data collection**	
Space group	P 61
Cell dimensions	
a, b, c (Å)	61.3, 61.3, 167.5
α, β, γ(˚)	90.0, 90.0, 120.0
Resolution (Å)	50.00 - 2.58 (2.67 - 2.58)
Rmeas (%)	16.5 (115.8)
Rpim (%)	5.5(46.8)
I/ δ I	2.45 (2.58)
Completeness (%)	59.66 (9.96)
Redundancy	8.0 (4.2)
**Refinement Statistics**	
Resolution (Å)	26.860 - 2.591 (2.684 - 2.591)
Reflections used	
Refinement	6592 (109)
R-free	317 (4)
R_{work}/R_{free}	0.239/0.260
Number of atoms	
Protein	2240
B-factors ($Å^2$)	
Protein	42
R.m.s. deviations	
Bond lengths (Å)	0.012
Bond angles (°)	1.79
Ramachandran plot	
Favored (%)	95.53
Allowed (%)	4.12
Outliers (%)	0.34

*Values in parentheses are for highest-resolution shell.

Supplementary Table S4. The interactions formed at the interfaces of M1R-M1R01 complex.

**M1R**	**M1R-01**
I38	L101
<u>Y42</u>	<u>N31</u>
R44	I28, N31, F32
<u>A94</u>	D105, L106, <u>A107</u>
L95	L106
<u>N96</u>	A107, E108, <u>Y110</u>
Y111	L106
T115	W100
<u>S119</u>	<u>F47</u>
A120	Y37, F47
V121	W100
D123	F47, Y59
<u>N124</u>	<u>Y37</u>, F47, A50
K125	Y59
L126	D33, A50, I51, S52, S57, T58, Y59
I128	D33
<u>N130</u>	<u>D33</u>, <u>D53</u>, L101, <u>S102</u>
V131	L101
<u>I132</u>	N31, <u>L101</u>, S102, I103, P104
I133	P104
<u>D134</u>	<u>P104</u>, D105
F148	L101
N157	L101
C158	W100, L101
K161	W100, L101, I103, L106
<u>Q165</u>	P104, <u>D105</u>, L106

A distance of cut-off 4 Å was used. Residues with underlines indicate the ones forming hydrogen bonds.
